# An ideal blood mimicking fluid for doppler ultrasound phantoms

**DOI:** 10.4103/0971-6203.29198

**Published:** 2006

**Authors:** H. Samavat, J. A. Evans

**Affiliations:** Dept. of Medical Physics, Hamedan University of Medical Science, Hamedan, Iran; * Dept. of Medical Physics, Leeds General Infirmary, Leeds, UK

**Keywords:** Blood mimicking fluid, doppler ultrasound, flow test object

## Abstract

In order to investigate the problems of detecting tumours by ultrasound it is very important to have a portable Doppler flow test object to use as a standardising tool. The flow Doppler test objects are intended to mimic the flow in human arteries. To make the test meaningful, the acoustic properties of the main test object components (tissue and blood mimic) should match closely the properties of the corresponding human tissues, while the tube should ideally have little influence. The blood mimic should also represent the haemodynamic properties of blood. An acceptable flow test object has been designed to closely mimic blood flow in arteries. We have evaluated the properties of three blood mimicking fluid: two have been described recently in the literature, the third is a local design. One of these has emerged as being particularly well matched to the necessary characteristics for *in-vitro* work.

For evaluating the performance of Doppler equipment, blood itself might appear to be the best fluid to use. However, there are a number of difficulties associated with using blood and its components. It is a potential biohazard and care must be taken to minimize this risk. The shelf life of blood is limited and erythrocytes in vitro are easily damaged. This prevents the use of blood as a standard fluid in long term studies of measurements and quality control. The rheological and acoustical properties of blood are likely to be different at room temperature than at 37°C and it is an additional complication to run flow models at the higher temperature.

Human blood is non-newtonian, which means that the viscosity is strongly dependent on shear rate. The dependence on shear rate is thought to occur in small vessels. In large vessels, it is acceptable to consider that blood is newtonian.

A blood-mimicking fluid (BMF) used in Doppler ultrasound (*invitro*) should ideally have similar properties to real blood. The ultrasound scanner would then receive an equivalent echo signal to that from blood.

Details of relevant acoustical properties of BMF must be considered carefully. Speed of sound, backscattered power, attenuation and also density, viscosity and particle size are important factors which should be examined in primary stage of manufacturing a BMF. Having a correct speed of sound is necessary in applications such as volume flow measurements where the vessel cross sectional area is being measured. The backscatter power is affected by shear rate, haematocrit and by turbulence.[1] Attenuation is also one of the parameters which will affect the relative weighting of Doppler signals from various parts of the vessel lumen. In real blood the attenuation is small (0.9 dB cm^−1^ at 3.5 MHz)[1] and it should not therefore be significant in any blood analogue.

The important rheological parameters are the density, viscosity and the particle concentration or haematocrite.

This paper describes a BMF in which the scatters are nylon particles of similar dimensions to erythrocytes and which closely models the properties of real blood.

## Materials and Methods

### Preparation of BMF

Three different kinds of BMF were selected for experimental study; one of them (sBMF) which seems to be more reliable in mimicking blood was studied in more details.

In sBMF ultrafine polyamide particles (Orgasol ELF Atochem, Paris) of diameter 5 mm, were used as the scattering particles. The Orgasol particles had a specified nominal density of 1.03 g cm^−3^. The sBMF was prepared by mixing the required amount of Orgasol (1.82%) with a fluid base of (% weight): pure water (83.86%); pure glycerol (10.06%); Sigma D4876 dextran of average molecular weight 185000 Dalton (3.36%); ICI synperonic N surfactant(0.9%). The sBMF was filtered through a 30 mm sieve to remove any residual clumps. The fluid base water/glycerol proportion principally determined the speed of sound of the sBMF and the fluid density. The density of sBMF was matched to the nylon particle density so that the particle was naturally buoyant. The dextran increased the viscosity of the sBMF and had no significant effects on other physical or acoustic properties. Surfactant was used to wet the Orgasol particles. The sBMF was easy to prepared and the typical preparation time was 4-5 h.

**Figure 1:**
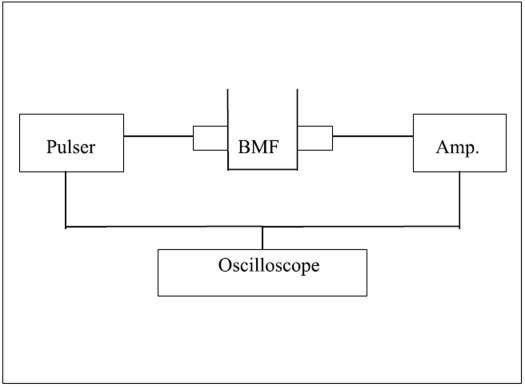
Block diagram of experimental set up for measurement in our lab.

After preparing, two important acoustic properties (velocity and attenuation) were measured. The sBMF was placed in a Plexiglas container of size 15 × 15× 15 cm. Two 5 MHz transducers (Acuson L558) were located face to face in two opposite side of the container, one of them as a transmitter and one of them as a receiver [Figure 1]. The container was filled with the sBMF (or distilled water); before each set of measurements the sBMF (or water) was degassed by using a vacuum pump.

For evaluating the system before measuring the sound properties of sBMF, the sound velocity of water in our experimental set up device was measured.

The speed of sound (SS) can be calculated using following equation:

Cm=d/t

With C_m_ = SS in the material inside the water, d = thickness of material and t = time.

Velocity measurement of water was repeated several times with checking temperature of fluid at time (to determine maybe even try heating it up to see if velocity changes a lot with temperature). Also attenuation measurements have been done in water to see what the losses are from the walls of the Plexiglas container.

Then the acoustic velocity in sBMF was measured, the comparison of our experience with results of the original study shows that both results are similar and are in recommended range of IEC [Table 1].

**Table 1:** Comparison of sound velocity in m s^−1^ and attenuation in dB cm^−1^ MHz, in water and selected BMF and references BMF

	*Ref. water 22°C*	*Ref. water 37°C*	Water* 22°C	Water* 37°C	*Ref. BMF 22°C*	BMF* 22°C
Average speed of sound(m/s)	1523.5 ± 0.5	1489.2 ± 0.5	1537 ± 5	1498.75 ± 5	1548 ± 5	1546 ± 5
Attenuation	**	**	**	**	0.05	0.06

* The measurements of this study

** Too small to be measured, BMF - Blood mimicking fluid

### Experimental flow model

Flow phantoms may provide steady or pulsatile flow. The purpose of the design should be considered in advance. In designing a flow phantom; the type of fluid, tubing and pump are very important and should be selected to have realistic velocity and attenuation values.

A preliminary steady flow model has been built and calibrated, using both constant pressure with no flow and constant pressure with flow with a range of tube sizes.

Many of the different particles which have been used in different blood mimicking fluids have density more than the red blood cells (RBC) which is undesirable. The fluid should have the same haemodynamic properties as well as physical properties of human blood. It is likely that the most critical for BMF is backscatter and its value for Orgasol is very closely to the IEC standard [Table 2].

**Table 2:** Physical and acoustic properties of selected blood mimicking fluid compared to whole human blood.[2]

*Properties*	*IEC 1685 Draft specification*	*Human blood 37°C*	*Selected BMF 22°C*
Scatterer		RBC	Orgasol ™(nylon)
Scatterer size (mm)	1050 ± 40	7	5
Hematocrit (% volume)	4 ± 0.4	45	<5
Density (kg m^−3^)	1570 ± 30	1053	1037 ± 2
Viscosity (mPa s)	< 0.1	3	4.1 ± 0.1
Velocity (ms^−1^)	Comparable to RBC	1583	1548 ± 5
Attenuation(dB/cm MHz)	Newtonian	0.15	0.05 ± 0.01
Backscatter (dB)		0	Comparable to RBC
Fluid properties		non Newtonian	Newtonian

BMF - Blood mimicking fluid, RBC - Red blood cells

The attenuation and velocity of selected BMF was measured in our lab for setting up the same condition of the original study and preventing of any systematic error. Figure 1 shows the block diagram of experimental set up for measurement of attenuation and velocity in selected BMF and water.

Among the papers that introduced the different kinds of BMFs, the BMF which is introduced[2] has been selected (sBMF) and prepared based on their recipe and some modification has been done as following:

By preparing dextran to Sigma D5501, sBMF_2_ with a higher viscosity was prepared.

sBMF_3_ with an antifungal agent (NaN_3_) was also prepared.

### Tube

An ideal tube material should have acoustic impedance close to fluid and vessels wall as well as good elasticity. Also it should be easily formed and available in various in diameters and with uniform wall thickness. In phantom the acoustic properties should be similar to BMF with the same physical properties with no extra sound reflection. Most of the systems designed for checking the accuracy of flow measurement have been made to facilitate calibration checks. Therefore relatively large vessels and flow rates are used throughout. In this study much smaller vessels and flows will be needed. The acoustic properties of different tube materials have been summarized in Table 3.

**Table 3:** Acoustic properties of three different tube materials in comparison with plasma.[3]

*Material*	*Density kgm^−3^*	*Velocity ms^−1^*	Impedance* Kg (s.m^2^)^−1^
Polyethylene	980	540	1.76 × 10^6^
Acrylic	1190	1330	3.27 × 10^6^
Glass (Pyrex)	2240	3280	1.26 × 10^7^
Plasma	1026	1546	~ water*

* Impedance of water at 25 °C = 1.49 × 10^6^ kg/s.m^2^

### Pump

Two different type of flow phantom have been described in the literature:[3]

- constant flow phantom
- pulsatile flow phantom

A pulsatile flow system, provides a more realistic model of arterial flow but it makes the interpretation of the resulting Doppler signal difficult,[4] because velocities and velocity profiles may not be known. A constant flow phantom provides a most reliable but less realistic option.

It is important to isolate the pump from the flow model in order to minimise vibration of the tubing. For producing stable flow in smallest vessels a small pump with large length of vessels is needed (i.e.: pump delivering ~0.400 ml per revolution).[5]

The pump which has been used is (EHEIM Nr. 381. Germany) and the flow velocity was controlled by varying the speed of the pump with a potentiometer, so the pump delivery was collaborated for each pump setting.

## Results and Discussion

The calculation of sound velocity in distilled water and sound velocity and attenuation in the selected BMF is given in Table 1.[2,6]

The haemodynamic and physical properties of human blood, sBMF and IEC specification have been summarized in Table 2

The acoustic properties of three different tube materials in the market at the time of the study in compare with human blood (plasma) have been summarized in Table 3.

Comparing three different materials in Table 3, it seems that by considering all three categories, acoustic properties of polyethylene tube is similar to human blood and this is consistent with most published data.

**Figure 2:**
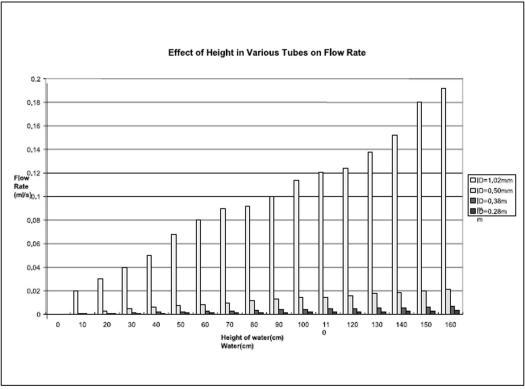
Measurement of fluid flow in different tube diameters and different height of water column.

**Figure 3:**
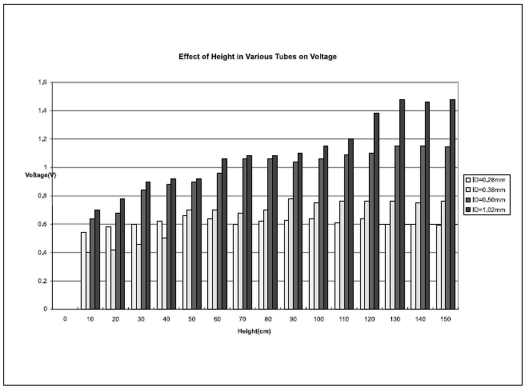
Measurement of voltage in pressure meter in different height of water column and various tube diameters.

After calibration of our flow model the flow rate in four tubes with different diameters was studied. The dependence of varying the pressure using a water column on the measured pressure and flow rate for a number of diameters is summarised in Figures 2 and 3.

Since both sBMF2 and sBMF3 were prepared with almost the original amount of glycerol (10-12%) there was no change in both densities. The other properties of BMF_1_ were not adjusted. The compositions of all three sBMFs and physical properties are given in Tables 4 and 5.

**Table 4:** Composition (components in mass %) of three BMFs

*Components*	*sBMF*	*sBMF_2_*	*sBMF_3_*
Water	83.86	84.02	82.04
Glycerol	10.06	10.8	12.06
Dextran 180 kD	3.36	3.36	--
Dextran 5-40 MD	--	--	1.15
Orgasol 5 mm	1.82	1.82	1.82
Synperonic N surfactant	0.9	0.9	0.90
NaN3	--	0.2	--

BMF - Blood mimicking fluid

**Table 5:** Physical properties of three sBMFs in 22°C

*Physical properties*	*sBMF*	*sBMF_2_*	*sBMF_3_*
Density (kg m^−3^)	1037 ± 2	1038 ± 2	1039 ± 2
Dynamic viscosity (mpa s)	4.1 ± 0.1	3.83 ± 0.1	4.7 ± 0.1
Sound velocity (m s^−1^)	1548 ± 5	1551 ± 5	1563 ± 5
Attenuation dB (cm. MHz)^−1^	0.05 ± 0.01	0.05 ± 0.01	0.06 ± 0.01
Back scattering (dB)	−36.0	−35.3	−36.7

BMF - Blood mimicking fluid

It can be seen from the measurements in different setting the BMF_3_ which has been designed in our lab achieves a close match to the necessary properties of blood for *in-vitro* work. However the ideal BMF has not yet been produced, differences in parameter values and physical behaviour still exist between the BMF and real blood in *in-vivo* studies.

## Conclusion

A flow test object has been developed with various options for the BMF. This test object provides flow situation that can be used to assess the capabilities of Doppler flow system. The physical properties of the studied BMF is very similar to IES standard. The BMF is easy to prepared and with adding antifungal can be used in acceptable duration of time (about 150 days) with almost no effect on physical and acoustical properties. In conclusion after modification, the BMF proved satisfactory for experimental work on Doppler ultrasound and suitable for the assessment of Doppler performance in a ultrasound system in agreement of the international standards.
